# New Therapeutic Approaches for the Treatment of Rheumatoid Arthritis may Rise from the Cholinergic Anti-Inflammatory Pathway and Antinociceptive Pathway

**DOI:** 10.1100/tsw.2010.207

**Published:** 2010-11-16

**Authors:** Xiao Hua Pan, Jianxin Zhang, Xiaowei Yu, Ling Qin, Ligeng Kang, Peng Zhang

**Affiliations:** ^1^ Department of Orthopaedics, Second Clinical Medical College, Jinan University, Shenzhen, Guangdong Province, China; ^2^ Department of Orthopaedics, Shandong University of Traditional Chinese Medicine, Jinan, Shandong Province, China; ^3^ Department of Orthopaedics, Second Affiliated Hospital, Nanjing Medical University, Nanjing, Jiangsu Province, China; ^4^ Center for Translational Medicine Research and Development, Shenzhen Institutes of Advanced Technology, Chinese Academy of Sciences, Shenzhen, Guangdong Province, China; ^5^ Department of Orthopaedics and Traumatology, Chinese University of Hong Kong, Hong Kong SAR, China; ^6^ Department of Orthopaedics, Taian Hospital of Traditional Chinese Medicine, Taian, Shandong Province, China

**Keywords:** rheumatoid arthritis, anti-inflammatory pathway, antinociceptive pathway, nicotinic acetylcholine receptor

## Abstract

Due to the complex etiology of rheumatoid arthritis (RA), it is difficult to be completely cured at the current stage although many approaches have been applied in clinics, especially the wide application of nonsteroidal anti-inflammatory drugs (NSAIDs) and disease-modifying antirheumatic drugs (DMARDs). New drug discovery and development via the recently discovered cholinergic anti-inflammatory and antinociceptive pathways should be promising. Based on the above, the nicotinic acetylcholine receptor agonists maintain the potential for the treatment of RA. Therefore, new therapeutic approaches may rise from these two newly discovered pathways. More preclinical experiments and clinical trials are required to confirm our viewpoint.

## INTRODUCTION

Rheumatoid arthritis (RA) is a chronic, systemic, inflammatory disorder that may affect many tissues and organs, which often leads to the destruction of articular cartilage and ankylosis of the joints[1]. Although the etiology of RA is still unknown, inflammation is the main pathological characteristic[2]. Consequently, to inhibit inflammation is of primary importance for the treatment of RA[3]. Painkillers and anti-inflammatory drugs have been widely used to relieve the symptoms of RA in clinics, especially the nonsteroidal anti-inflammatory drugs (NSAIDs) and disease-modifying antirheumatic drugs (DMARDs),

*These authors contributed equally to this paper as first author.
**Corresponding author.

which are used to inhibit or halt the underlying immune process and prevent long-term damage[4]. Nonetheless, the RA patient cannot be cured via simple treatment. Consequently, new therapeutic methods for RA are urgently needed. Based on our previous studies related to vagus nerve stimulation (VNS)[5] and the current literature review, we believe that the newly discovered cholinergic anti-inflammatory pathway and the antinociceptive pathway are potential targets for RA treatment .

## THE PROINFLAMMATORY CYTOKINES PLAY ESSENTIAL ROLE IN RA DEVELOPMENT

Proinflammatory cytokines play a decisive role in the generation of the inflammatory and destructive response[6]. More importantly, tumor necrosis factor alpha (TNF-α) is now targeted in the treatment of RA, and an antibody of TNF-α has proved to be effective[7]. Other cytokines are being tested and treated as targets for RA therapy with promising results[8]. All the above has confirmed the importance of these proinflammatory cytokines in the pathological process of RA from the reverse side. It has been widely accepted that cytokines are involved in each phase of the pathogenesis of RA, by promoting autoimmunity (during the prearticular phase), by maintaining chronic inflammatory synovitis, and by driving the destruction of joints[9]. Among all the proinflammatory cytokines, TNF-α is the major one that presents in the rheumatoid joint and induces the synthesis and secretion from synovial fibroblasts of matrix-degrading proteases, interleukin-6 (IL-6), interleukin-8 (IL-8), and granulocyte-macrophage colony stimulating factor (GM-CSF)[10]. It has been found that TNF-α exerts its effects through binding to two receptors: the type I (p55) and type II (p75), which are found on immune, inflammatory, and endothelial cells[11]. More importantly, Lam et al. discovered that TNF-α induced osteoclastogenesis by directly targeting macrophages in the presence of permissive levels of ligand for the receptor activator of NF-κB (RANKL), which may contribute to joint cartilage and bone destruction[12]. With further in-depth investigation, many factors have been discovered that may play important roles in TNF-α–induced osteoclastogenesis, e.g., TRAF2[13], IL-12[14], IL-7, IL-18[15], and IL-4[16]. Some of them are indispensable for activation of this process, while the others may have the opposite effect and inhibit osteoclastogenesis. Besides TNF-α, proinflammatory cytokines such as IL-1[17], IL-16[18], and high-mobility group B (HMGB)[19] play important roles as well. Therefore, a decreased inflammatory cytokine level may lead to ameliorated symptoms of RA. Correspondingly, an inflammatory cytokine–targeted therapeutic approach is increasingly appreciated.

## PAIN IS AN IMPORTANT SYMPTOM OF RA

Most RA patients suffer from pain as well as pain-related motion dysfunction. Actually, besides the inflamed joints, other normal tissues may be involved. Until now, the neurophysiological mechanisms underlying pain in RA remained unclear, while experimental models of inflammatory arthritis suggest that changes of neuronal sensitivity at both peripheral and central levels may be important[20]. When RA occurs, within the joint, inflammation will inevitably lead to the activation of articular sensory receptors[21]. Moreover, Morris et al. reported that enhanced sensitivity of a population of sensory fibers partly contributed to the hyperalgesia of RA[22]. Since pain may lead to a series of complications and physical problems that will worsen the RA patients' condition, how to relieve pain has become another important issue. NSAIDs have been used as a therapeutic routine for the treatment of inflammation-induced pain in clinics. Side effects such as nausea, vomiting, diarrhea, constipation, decreased appetite, rash, dizziness, headache, drowsiness, etc. are inevitable. Opioids are another choice. It has been recognized that opioids mediate pain relief solely via centrally located opioid receptors in the brain and spinal cord, as well as by activating opioid receptors on peripheral sensory neurons[23]. Moreover, peripheral opioid receptor agonists have been found to inhibit inflammation and improve wound healing[24,25]. Therefore, opioids are frequently prescribed analgesics for patients suffering from rheumatic disease–induced pain, including

RA[26]. The unwanted side effects, e.g., nausea, constipation, dizziness somnolence, and vomiting, greatly limit their application as well. Consequently, it is necessary to explore new drugs for the treatment of inflammation-induced pain.

## HOW DO THE CHOLINERGIC ANTI-INFLAMMATORY AND ANTINOCICEPTIVE PATHWAYS WORK?

The ‘‘cholinergic anti-inflammatory pathway’’, first reported by Tracey[27], is recognized as the efferent arm of the inflammatory reflex. In the preliminary research, the cholinergic anti-inflammatory pathway was activated by direct electrical stimulation of the efferent vagus nerve, which may inhibit the synthesis of TNF in liver, spleen, and heart, and attenuate serum concentrations of TNF during endotoxemia[28]. With more in-depth study, the $\alpha_7$ nicotinic acetylcholine receptor ($\alpha_7$ nAChR) was found to be the molecular dovetail between the cholinergic nervous system and the innate immune system[29], which mainly expresses on the membrane of immune cells, including monocytes, macrophages, T and B lymphocytes, and dendritic cells[16]. Moreover, in RA patients, $\alpha_7$ nAChR was found in the inflamed synovia[30]. Simultaneously, it has been reported that tissue macrophages, but not circulating monocytes, produce most of the TNF during an excessive inflammatory response[27]. Therefore, most of the recent research focused on the effect of $\alpha_7$ nAChR on the activity of macrophages. When the cholinergic receptor, which lies on the membrane of the macrophage, combines with its ligand, the synthesis of proinflammatory cytokines (TNF-α, IL-1, and IL-18), but not anti-inflammatory cytokines (such as IL-10), will be inhibited[28]. During this process, signaling transduction pathways, such as JaK2-STAT3, were involved[31,32]. In detail, nicotine may activate nAChRs, and Jak2 was recruited to the $\alpha_7$ subunit of the nAChR and was phosphorylated after nicotine binding, leading to phosphorylation of STAT3. Moreover, Niziri et al. reported the immune modulation effect of $\alpha_7$ nAchR via suppression of Th1 and Th17 responses, which even deepened the current mechanism research[33]. It is interesting that $\alpha_4\beta_2$ nAChR was reported to share a similar anti-inflammatory function as $\alpha_7$ nAChR, which broadens our knowledge concerning the cholinergic anti-inflammatory pathway[34].

Besides the anti-inflammatory effect, the nAChRs have been found to have antinociceptive efficacy[35], which has been proven by a variety of species and pain tests[36]. Further studies indicated that nAChRs are expressed in a variety of CNS loci, e.g., medulla[37], midbrain[38], the thalamus, and the pedunculopontine tegmental nucleus[39]. Both $\alpha_4\beta_2$ and $\alpha_7$ nAChRs are essential subtypes contributing to the cholinergic antinociceptive efficacy. Rowley et al. reported that nicotine, which is regarded as an agonist of $\alpha_4\beta_2$ and $\alpha_7$ nAChRs, exerts antinociceptive efficacy in a mouse model of postoperative pain via reducing nociceptive input to the superficial and deep dorsal horn[40]. From the opposite side, blocking $\alpha_7$ nAChR using α-BGTX resulted in decreased antinociceptive effects of $\alpha_7$ nAChR agonists, e.g., DMXB and 4-OH-DMXB l[41]. Therefore, nAChRs are the main factor that contributes to cholinergic anti-inflammatory and antinociceptive effects.

## WHAT HAS BEEN DONE AND NEEDS TO BE DONE

Rheumatologists and orthopedic surgeons have turned their vision toward the treatment of RA using the cholinergic anti-inflammatory pathway[42,43,44,45]. More importantly, $\alpha_7$ nAChR was found in the synovial tissue of RA patients, which even increased the feasibility of curing RA by activating the cholinergic anti-inflammatory pathway[30,46]. As to the therapy of RA, to our current knowledge, no report was found using the antinociceptive pathway, which should be strengthened in the future research. On the other hand, clinical proof-of-principle studies will be required to determine if nAChR agonists are active in pathological pain and inflammation conditions. We are pleased to know that such work is in process. A clinical trial entitled “*Anti-Inflammatory Effects of GTS-21 After LPS*” has been ongoing since October 30, 2008 (http://clinicaltrials.gov/ct2/show/NCT00783068). However, more clinical trials are to be expected after strictly designed *in vivo* and *in vitro* experiments obtain ideal results. That is our struggle in the future.

## PROSPECT

Based on the above analysis, nicotinic cholinergic receptors are potential therapeutic targets for the treatment of RA via activation of cholinergic anti-inflammatory and antinociceptive pathways (Fig. 1). Therefore, the agonists of nicotinic cholinergic receptors are expected to contribute to the symptom amelioration of the RA patient. To achieve this goal, as we have mentioned in our previous paper, a three-step strategy should be considered[47]. First, the best candidate of nAChR agonists should be selected and the therapeutic effect should be confirmed. Second, the preclinical experiments should be performed. Third, strict phase I to phase III clinical trials are required that will provide stronger evidence for the potential application of such drugs in the future.

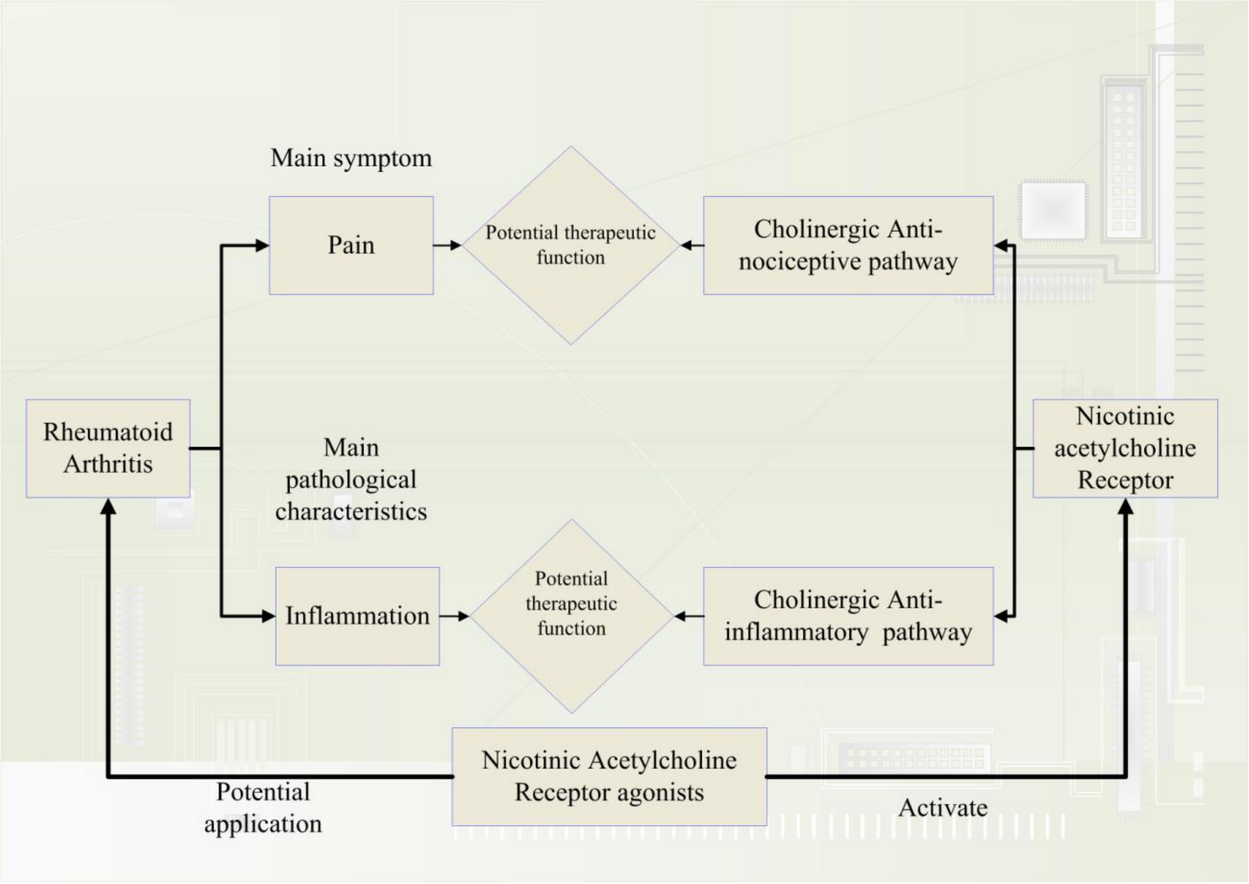

**FIGURE 1**. The diagrammatic illustration of the potential application of cholinergic anti-inflammatory and antinociceptive pathways.

## ACKNOWLEDGMENT

This research was supported by the National Natural Science Foundation of China. Support number: 81000786.
